# Prefecture government fiscal intervention and corporate asset allocation: The perspective of transaction cost theory

**DOI:** 10.1371/journal.pone.0345478

**Published:** 2026-07-01

**Authors:** Jieyang Pan

**Affiliations:** School of Economics, Faculty of Arts and Social Sciences, The University of Sydney, Sydney, NSW, Australia; Universiti Malaysia Sabah, MALAYSIA

## Abstract

This study examines the impact of prefecture-level government fiscal intervention on corporate asset allocation in China from the perspective of transaction cost theory. Using a panel dataset of Chinese listed firms from 2008 to 2023, we find that increased fiscal intervention significantly reduces firms’ allocation to operating assets while increasing financial asset holdings, indicating a substitution effect. Mechanism analysis suggests that fiscal intervention raises regional transaction costs, discouraging long-term productive investment. Heterogeneity analysis shows stronger effects among private firms and firms more dependent on local economies. These findings provide new evidence on how government intervention shapes firm-level resource allocation and offer policy implications for improving fiscal efficiency and supporting real-sector development in emerging economies.

## 1. Introduction

Corporate asset allocation decisions are central to firm performance and economic growth, as they determine the balance between productive investment and financial activities [1,2]. Understanding what drives these allocation decisions has long been a core concern in corporate finance and industrial organization [3,4]. At the aggregate level, firms’ production activities fuel economic growth by driving technological progress, industrial upgrading, and structural transformation [5,6].

Governments play a critical role in shaping these decisions through fiscal policies that influence market conditions, incentives, and institutional environments [7,8]. Fiscal policy, through public expenditure, taxation, and subsidies, directly and indirectly influences resource allocation, market conditions, and corporate incentives [9,10]. While fiscal intervention can promote investment through infrastructure and subsidies, excessive or inefficient intervention may distort resource allocation, increase uncertainty, and crowd out productive investment. This issue is particularly relevant in emerging economies such as China, where local governments exert substantial influence over economic activities [11–13].

The existing literature documents two competing views of how fiscal intervention affects corporate investment. On one hand, well-designed fiscal policies, such as infrastructure spending, education investment, and R&D subsidies, can enhance private-sector productivity, reduce operational costs, and “crowd in” corporate investment in operating assets [14–18]. Fiscal incentives such as R&D tax credits can stimulate accumulation of specific operating assets, especially where positive externalities exist [19–21]. On the other hand, excessive or poorly designed intervention may generate the opposite effect. Large-scale spending funded through distortionary taxation can “crowd out” private investment by raising the cost of capital or reducing after-tax returns [22–24]. Government intervention can also misallocate resources, particularly when politically motivated lending or procurement favors less productive firms [25–28]. Moreover, policy uncertainty stemming from unpredictable fiscal adjustments may suppress irreversible long-term investments [29–32].

China offers a rich empirical setting for studying this question. Its capital markets have expanded rapidly, yet the institutional framework remains less developed relative to the pace of economic growth, creating conditions in which government intervention can generate significant distortions. A substantial body of research has examined soft budget constraints for state-owned enterprises (SOEs), regional competition for growth, and political connections in China [33–36]. Prefecture governments are especially important in this context. They exercise significant authority over regional economic development and resource allocation [37], and their interventionist tendencies are reinforced by China’s “GDP growth-oriented” appraisal system. While this system has fueled rapid growth, it has also incentivized excessive intervention that can lead to inefficiency and structural imbalance [38–40].

This study investigates how prefecture-level fiscal intervention affects corporate operating asset allocation using a transaction cost framework. Coase [41] and Williamson [42–44] demonstrate that market transaction costs profoundly shape firms’ organizational boundaries, governance structures, and investment decisions. When external transaction costs are high, firms may internalize activities, choose less asset-specific investments, or forgo productive investments altogether [45,46]. Fiscal intervention by prefecture governments can raise transaction costs through several channels: policy instability, weakened contract enforcement, and increased rent-seeking opportunities [47]. Faced with higher transaction costs, firms have weaker incentives to commit resources to highly specific, illiquid operating assets and may instead shift toward more liquid financial assets or short-term projects with lower transaction intensity [48]. This mechanism may also contribute to corporate financialization [49–52].

We argue that fiscal intervention alters the institutional environment, increasing transaction costs and discouraging long-term investment in operating assets. Using firm-level panel data from 2008–2023, we provide empirical evidence that fiscal intervention reduces operating asset allocation and increases financialization. This study contributes to the literature by linking government intervention to firm-level asset allocation through transaction cost mechanisms.

This paper makes three main contributions. First, it extends the literature on the economic consequences of prefecture government fiscal intervention. Previous studies have focused on aggregate investment levels [53,54]. We instead examine how fiscal intervention reshapes the composition of firm-level asset, specifically, the allocation between operating and financial assets. Second, it enriches research on the determinants of corporate asset allocation by introducing a transaction cost perspective. Our mechanism analysis demonstrates that fiscal intervention influences firms’ asset allocation by raising external transaction costs, providing micro-level evidence on how government behavior transmits to corporate decisions. Third, the study provides systematic empirical evidence from China’s unique institutional context, where prefecture governments hold substantial economic power. Our findings complement cross-country studies [55,56] and offer policy insights not only for China’s fiscal reform but also for other emerging and transition economies where subnational government intervention shapes corporate behavior.

The paper proceeds as follows. Section 2 describes the research design, including model specification, data, and variable definitions. Section 3 presents baseline results, instrumental variable estimates, and robustness checks. Section 4 examines the link between fiscal intervention and corporate financialization. Section 5 investigates the transaction cost mechanism. Section 6 reports heterogeneity analyses. Section 7 concludes with policy implications.

## 2. Research design

This section outlines our empirical strategy for identifying the effect of prefecture government fiscal intervention on corporate operating asset allocation.

### 2.1 Model specification

To estimate the effect of fiscal intervention on corporate asset allocation, we employ a firm-level fixed-effects model controlling for time-invariant firm heterogeneity and macroeconomic shocks. All control variables are lagged by one period to mitigate reverse causality. Standard errors are clustered at the firm level to account for serial correlation. We estimate the following fixed-effects model:


$$\begin{array}{c} {Operating\_Assets}_{ijt}=\alpha_{\text{0}}+\beta {\cdot Intervene}_{jt}+X_{it-\text{1}}\overset{\text{'}}{\underset{\text{1}}{\theta }}{+X}_{jt-\text{1}}\overset{\text{'}}{\underset{\text{2}}{\theta }}+\eta_{i}+\mu_{t}+\varepsilon_{ijt} \end{array}$$ (1)

where *i*, *j* and *t* correspond to the firm, city, and year, respectively. ${Operating\_Assets}_{ijt}$ measures the firm’s operating asset allocation. ${Intervene}_{jt}$ captures the degree of prefecture government fiscal intervention. $X_{it-\text{1}}$ is a vector of firm-level control variables, including indicators of financial condition, governance structure, and growth opportunities. $X_{jt-\text{1}}$is a vector of city-level control variables, covering macroeconomic factors affecting regional economic development and the business environment. All control variables are lagged by one period to mitigate reverse causality. $\eta_{i}$ and $\mu_{t}$ control the firm-level and year-level fixed effects, respectively. $\varepsilon_{ijt}$ is the error term, representing other unobserved factors in the model. Standard errors are clustered at the two-digit industry level to account for within-industry correlation. All estimations are performed using Stata 17.0 with the *reghdfe* package. The coefficient of interest is β, the coefficient of ${Intervene}_{jt}$, which captures the effect of ${Intervene}_{jt}$ on ${Operating\_Assets}_{ijt}$. A significantly negative β indicates that higher fiscal intervention reduces firms’ operating asset holdings.

To address endogeneity concerns, we apply the heteroskedasticity-based instrumental variable approach [57,58], which generates internal instruments without requiring external data. This method relies on model-based identification through heteroskedastic error structures.

### 2.2 Data source and sample processing

Our sample comprises industrial firms listed on the Shanghai and Shenzhen stock exchanges from 2007–2023. Industrial firms typically hold a high proportion of operating assets, making their asset allocation decisions directly relevant to the real economy. We apply the following selection criteria:

(1) Firms designated as *ST* (Special Treatment) or ST^*^ (Special Treatment*) are excluded. Their unstable operating conditions may distort normal asset allocation decisions.
(2) Insolvent firms (debt-to-asset ratio is greater than 1) are excluded. Their asset allocation reflects distress-related factors rather than strategic choices.
(3) Observations with missing data for key variables are excluded.

Firm-level financial data come from the CSMAR database, and the macroeconomic data come from the National Bureau of Statistics of China. The final sample consists of 23,531 firm-year observations. All continuous variables are winsorized at the 1st and 99th percentiles to mitigate outlier effects.

### 2.3 Variable definitions

#### 2.3.1 Key explanatory variable.

We measure prefecture government fiscal intervention (*Intervene*) as the ratio of prefecture-level fiscal budgetary expenditure to GDP. This indicator captures the intensity with which the prefecture government deploys economic resources through fiscal channels. Scaling by GDP accounts for the size of the regional economy and enables cross-city comparisons. A higher ratio implies greater government control over regional resource allocation.

#### 2.3.2 Dependent variable.

We define operating asset allocation (*Operating_Assets*) as the sum of fixed assets, intangible assets, and other long-term assets, divided by period-end total assets [50]. These three categories share two important features: they are long-term by nature, so allocation decisions reflect strategic assessments of the future business environment; and they tend to be highly asset-specific, illiquid, and costly to dispose of, making firms more cautious and more sensitive to external conditions when allocating resources to them.

#### 2.3.3 Control variables.

This paper incorporates a set of control variables to capture firms’ fundamental characteristics. Specifically, we include proxies for operating conditions: the debt-to-asset ratio (*Lev*), growth opportunities (*Growth*), firm size (*Size*), and return on assets (*ROA*). Corporate governance structure is captured by the ownership concentration of the largest shareholder (*Top*1), management shareholding ratio (*Msh*), board size (*Board*), and institutional investor shareholding ratio (*Ins*).

To control for the effects of regional economic characteristics, we include the logarithm of prefecture per capita GDP (*Lnpergdp*), the share of secondary industry in GDP (*Industry*), the degree of credit expansion (*Debt*), the Fan Gang marketization index score (*Market*), and the level of total retail sales of social consumer goods (*Sale*). By controlling for these variables, we can more accurately test the impact of prefecture government fiscal intervention on corporate operating asset allocation, mitigating omitted variable bias. Definitions of main variables are shown in Table 1. Table A1 in the Appendix reports the correlation matrix.

**Table 1:** Definitions of main variables.

	Variable	Definition	Calculation Method
Explained Variable	*Operating Assets*	Corporate operating asset allocation	The ratio of the sum of fixed assets, intangible assets, and other long-term assets to period-end total assets.
Explanatory Variable	*Intervene*	Prefecture government fiscal intervention	The ratio of prefecture fiscal budgetary expenditure to GDP.
Firm-Level Control Variable	*Lev*	Debt-to-asset ratio	The ratio of debt to period-end total assets.
	*Growth*	Investment opportunities	The growth rate of total operating revenue.
	*Size*	Firm size	The logarithm of period-end total assets.
	*ROA*	Return on assets	The ratio of net profit to period-end total assets.
	*Top1*	Ownership concentration	The ratio of shares held by the largest shareholder to the total number of outstanding shares.
	*Msh*	Management shareholding ratio	The ratio of shares held by directors, supervisors, and senior management to the total number of outstanding shares.
	*Board*	Size of board	The logarithm of the total number of board members.
	*Ins*	Institutional investor shareholding	The ratio of shares held by institutional investors to the total number of outstanding shares.
Prefecture-Level Control Variable	*Lnpergdp*	Logarithm of per capita GDP	The logarithm of prefecture per capita GDP.
	*Industry*	Proportion of secondary industry	The ratio of the gross output value of the secondary industry to GDP.
	*Market*	Marketization level	The overall score of the Fan Gang marketization index.
	*Debt*	Credit expansion	The ratio of the period-end balance of loans to the period-end balance of deposits in the financial institution.
	*Sale*	Retail sales of consumer goods	The logarithm of the total retail sales of social consumer goods in the prefecture.

### 2.4 Descriptive statistics

Table 2 presents the descriptive statistics. The mean of *Operating_Assets* is 0.283 (SD is 0.159), indicating substantial cross-firm variation in operating asset allocation. For *Intervene*, the mean is 0.149 (SD is 0.050, range from 0.055 to 0.393), reflecting large regional disparities in fiscal intervention across Chinese prefectures. In some regions, market forces play a more dominant role, while in others, governments intervene more heavily. This variation provides a solid empirical foundation for identifying the effect of fiscal intervention.

**Table 2:** Descriptive statistics of main variables.

Variable	Observations	Mean	Std. Dev.	Min	Max
*Operating_Assets*	23,531	0.283	0.159	0.029	0.735
*Intervene*	23,531	0.149	0.050	0.055	0.393
*Lev*	23,531	0.410	0.198	0.051	0.865
*Growth*	23,531	0.169	0.350	−0.492	2.074
*Size*	23,531	22.080	1.261	19.870	25.980
*ROA*	23,531	34.510	14.730	8.899	74.017
*Top1*	23,531	13.599	19.966	0.000	68.591
*Msh*	23,531	8.591	1.724	5.000	15.000
*Board*	23,531	44.257	24.836	0.303	91.685
*Ins*	23,531	0.040	0.061	−0.221	0.199
*Lnpergdp*	23,531	8.730	1.137	5.131	10.674
*Industry*	23,531	0.423	0.112	0.158	0.718
*Market*	23,531	8.500	2.015	2.810	12.540
*Debt*	23,531	0.752	0.195	0.312	1.441
*Sale*	23,531	16.979	1.147	13.054	19.013

## 3. Main empirical results

### 3.1 Baseline regression

Table 3 reports the baseline results from estimating Equation (1). Column (1) includes only firm and year fixed effects. The coefficient on Intervene is −0.14962, significant at the 1% level. Column (2) adds firm-level controls; the coefficient becomes −0.17464, significant at the 1% level. Column (3) further includes city-level controls, yielding a coefficient of −0.21064, significant at the 1% level. The stability of the coefficient across specifications strengthens confidence in a genuine negative relationship.

**Table 3:** Baseline regression results.

Variable	Explained Variable: *Operating_Assets*		
	(1)	(2)	(3)
*Intervene*	−0.14962***	−0.17464***	−0.21064***
	(−2.82)	(−3.35)	(−3.64)
*Lev*		0.06735***	0.06805***
		(4.05)	(4.02)
*Growth*		0.00050	0.00050
		(0.20)	(0.21)
*Size*		−0.00780	−0.00778
		(−1.06)	(−1.05)
*ROA*		−0.21024***	−0.21087***
		(−8.33)	(−8.54)
*Top1*		0.00023	0.00022
		(0.93)	(0.89)
*Msh*		−0.00046***	−0.00048***
		(−3.67)	(−3.83)
*Board*		−0.00198	−0.00192
		(−1.31)	(−1.26)
*Ins*		−0.00026	−0.00026
		(−1.63)	(−1.62)
*Lnpergdp*			0.01195
			(1.05)
*Industry*			0.03424
			(0.78)
*Market*			−0.00322
			(−0.84)
*Debt*			0.02521
			(1.52)
*Sale*			−0.02520***
			(−2.77)
*Constant*	0.30492***	0.48878***	0.81103***
	(38.59)	(3.11)	(4.59)
Firm/Year-Level Fixed Effects	YES	YES	YES
*N*	23531	23531	23531
*adj. R* ^*2*^	0.741	0.750	0.751

Note: *, **, and *** denote significance at the 10%, 5%, and 1% levels, respectively. The t-statistics in parentheses are based on robust standard errors clustered at the two-digit industry level (industry codes). Control variables are lagged by one year.

The economic magnitude is meaningful. A one-standard-deviation increase in Intervene (0.050) reduces *Operating_Assets* by 0.01053 (0.050 × 0.21064), equivalent to 3.72% of the sample mean (0.283). To put this in perspective, this reduction is comparable to roughly one-quarter of the standard deviation of *Operating_Assets* (0.159), suggesting that fiscal intervention’s effect on asset allocation is economically as well as statistically significant.

Comparing across columns reveals an interesting pattern: the coefficient grows in absolute magnitude as controls are added. Adding firm-level controls strengthens the coefficient from −0.14962 to −0.17464, and adding city-level controls further amplifies it to −0.21064. This pattern suggests that omitting these controls would understate the negative impact of fiscal intervention, consistent with the presence of confounding factors that correlate positively with both fiscal intervention and operating asset allocation. For instance, more developed regions tend to have both lower fiscal expenditure ratios and higher corporate investment, so failing to control for regional development would bias the estimated effect toward zero.

The negative coefficient of fiscal intervention suggests that government involvement increases uncertainty and transaction costs, discouraging firms from committing to long-term productive assets. Instead, firms shift toward more liquid financial assets, which are less exposed to policy risks. This supports the hypothesis that institutional distortions influence corporate investment behavior.

### 3.2 Instrumental variable regression

Endogeneity may arise from two sources. First is the reverse causality. Firms’ aggregate asset allocation decisions within a region might influence the prefecture government’s fiscal expenditure. Second is omitted variables. Unobserved factors could correlate with both fiscal intervention and firms’ asset allocation.

To address these concerns, we employ the heteroscedasticity-based instrumental variable approach [57,58]. This method constructs internal instruments from the model’s heteroscedastic error structure without relying on external data or policy shocks. Table 4 reports the results. Across different kernel bandwidths, the coefficient on *Intervene* remains negative and significant at the 1% level, corroborating the baseline finding.

**Table 4:** Instrumental variable regression results.

Variable	Explained Variable: *Operating_Assets*	
	bandwidth = 3	bandwidth = 4
	(1)	(2)
*Intervene*	−0.31344***	−0.31517***
	(−3.45)	(−3.19)
*Lev*	0.06461***	0.06458***
	(6.27)	(5.72)
*Growth*	−0.01177***	−0.01175***
	(−3.58)	(−3.55)
*Size*	0.01659***	0.01661***
	(9.78)	(8.83)
*ROA*	−0.23616***	−0.23622***
	(−9.41)	(−8.87)
*Top1*	0.00115***	0.00115***
	(9.41)	(8.54)
*Msh*	−0.00087***	−0.00087***
	(−8.79)	(−8.05)
*Board*	0.00634***	0.00632***
	(6.66)	(6.02)
*Ins*	−0.00018*	−0.00018*
	(−1.94)	(−1.77)
*Lnpergdp*	−0.00646	−0.00635
	(−0.90)	(−0.81)
*Industry*	−0.03384	−0.03436
	(−1.06)	(−0.99)
*Market*	−0.00426***	−0.00425***
	(−4.47)	(−4.04)
*Debt*	−0.00600	−0.00602
	(−0.67)	(−0.61)
*Sale*	−0.01959**	−0.01973**
	(−2.55)	(−2.35)
*Constant*	0.31738***	0.31886***
	(3.38)	(3.12)
Firm/Year-Level Fixed Effects	YES	YES
*N*	23531	23531
*adj. R* ^*2*^	0.155	0.155

Note: *, **, and *** denote significance at the 10%, 5%, and 1% levels, respectively. The t-statistics in parentheses are based on robust standard errors clustered at the two-digit industry level (industry codes). Control variables are lagged by one year.

### 3.3 Robustness analysis

#### 3.3.1 Alternative measures of corporate operating asset allocation.

Operating assets consist mainly of fixed and intangible assets with distinct economic characteristics. Fixed assets serve as the physical foundation for firms’ production activities and are directly involved in value creation. Intangible assets, on the other hand, are key to firms’ differentiated competition, indirectly enhancing firms’ value-added through technological monopolies, brand premiums, or resource control. We separately regress the ratios of fixed assets (*Fixed_Assets*) and intangible assets (*Intangible_Assets*) on *Intervene*. The results are shown in Table 5. In columns (1) and (2), the coefficient for fiscal intervention is significant at the 1% level in both cases, indicating that the main conclusion remains robust to alternative dependent variables. A comparison of the coefficients in columns (1) and (2) reveals that the negative impact of prefecture government fiscal intervention is larger in magnitude for fixed asset allocation, suggesting that firms’ adjustments to tangible asset allocation decisions are more sensitive to external intervention.

**Table 5:** Robustness check results – alternative dependent variables.

Variable	Explained Variable: *Fixed_Assets*	Explained Variable: *Intangible_Assets*
	(1)	(2)
*Intervene*	−0.17476***	−0.03859***
	(−3.16)	(−3.03)
*Lev*	0.06534***	0.00334
	(4.12)	(0.66)
*Growth*	0.00079	−0.00045
	(0.35)	(−0.65)
*Size*	−0.00936	0.00135
	(−1.56)	(0.89)
*ROA*	−0.16786***	−0.04095***
	(−7.47)	(−6.16)
*Top1*	0.00035	−0.00004
	(1.64)	(−0.45)
*Msh*	−0.00039***	−0.00010
	(−3.21)	(−1.55)
*Board*	−0.00193	0.00027
	(−1.26)	(0.82)
*Ins*	−0.00028*	−0.00002
	(−1.76)	(−0.38)
*Lnpergdp*	0.01576	−0.00370
	(1.41)	(−0.99)
*Industry*	0.03271	0.00775
	(0.91)	(0.56)
*Market*	−0.00267	−0.00025
	(−0.83)	(−0.24)
*Debt*	0.02508	0.00017
	(1.64)	(0.04)
*Sale*	−0.02152**	−0.00252
	(−2.41)	(−0.86)
*Constant*	0.68857***	0.09688*
	(4.27)	(1.84)
Firm/Year-Level Fixed Effects	YES	YES
*N*	23531	23531
*adj. R* ^*2*^	0.741	0.750

Note: *, **, and *** denote significance at the 10%, 5%, and 1% levels, respectively. The t-statistics in parentheses are based on robust standard errors clustered at the two-digit industry level (industry codes). Control variables are lagged by one year.

#### 3.3.2 Exclusion of samples during financial crisis and COVID-19 pandemic periods.

The shocks of the financial crisis and the COVID-19 pandemic led to significant fluctuations in the macroeconomic environment. Firms likely faced tightened financing conditions, shrinking market demand, supply chain disruption, and pessimistic business expectation. These factors could cause corporate asset allocation behavior to deviate from patterns in the normal period, thereby biasing the empirical estimates. To mitigate the impact of these special events, we exclude samples from the 2007−2008 global financial crisis, the 2015 Chinese stock market turbulence, and the post-2020 COVID-19 period. The regression results after these exclusions are shown in columns (1)-(3) of Table 6. The results show that the coefficient for fiscal intervention remains significantly negative at the 1% level in all cases, indicating that the exclusion of these special period samples has little impact on the core regression results. In other words, the baseline conclusion is not driven by corporate asset allocation behavior during specific crisis periods or amid extreme macroeconomic shocks.

**Table 6:** Robustness check results – exclusion of special year samples.

Variable	Explained Variable: *Operating_Assets*		
	Exclude samples during 2007–2008	Exclude samples in 2015	Exclude samples after 2020
	(1)	(2)	(3)
*Intervene*	−0.16845***	−0.22373***	−0.20299***
	(−2.94)	(−3.92)	(−2.74)
*Lev*	0.07216***	0.06578***	0.07286***
	(4.06)	(3.98)	(3.62)
*Growth*	0.00009	0.00247	−0.00001
	(0.04)	(0.97)	(−0.01)
*Size*	−0.01136	−0.00666	−0.01361
	(−1.60)	(−0.90)	(−1.61)
*ROA*	−0.19643***	−0.21101***	−0.20972***
	(−7.47)	(−8.52)	(−7.49)
*Top1*	0.00034	0.00020	0.00050*
	(1.29)	(0.84)	(1.72)
*Msh*	−0.00044***	−0.00048***	−0.00069***
	(−4.05)	(−3.85)	(−3.74)
*Board*	−0.00214	−0.00190	−0.00160
	(−1.41)	(−1.24)	(−0.90)
*Ins*	−0.00030**	−0.00026	−0.00038*
	(−2.28)	(−1.52)	(−1.82)
*Lnpergdp*	0.00950	0.01227	0.01762
	(0.82)	(1.07)	(1.43)
*Industry*	0.05601	0.04181	0.00754
	(1.51)	(0.94)	(0.14)
*Market*	−0.00320	−0.00354	−0.00409
	(−0.79)	(−0.91)	(−0.94)
*Debt*	0.02835*	0.02446	0.01713
	(1.79)	(1.43)	(0.83)
*Sale*	−0.02096**	−0.02671***	−0.02281**
	(−2.35)	(−2.94)	(−2.29)
*Constant*	0.81626***	0.81085***	0.87107***
	(4.85)	(4.39)	(3.84)
Firm/Year-Level Fixed Effects	YES	YES	YES
*N*	22093	21954	17665
*adj. R* ^*2*^	0.765	0.748	0.761

Note: *, **, and *** denote significance at the 10%, 5%, and 1% levels, respectively. The t-statistics in parentheses are based on robust standard errors clustered at the two-digit industry level (industry codes). Control variables are lagged by one year.

#### 3.3.3 Exclusion of high-administrative-level city samples.

China’s urban system is characterized by a clear administrative hierarchy, with cities at different levels varying significantly in their resource access capability and policy-making authority. Generally, municipalities, sub-provincial cities, and provincial capitals tend to concentrate a greater amount of economic and political resources, giving their prefecture governments greater autonomy and stronger implementation capacity in intervening in economic activities and promoting regional development. In contrast, ordinary prefecture governments may face more constraints in terms of resource endowments and policy-making. To ensure our results are not driven by extreme macroeconomic shocks, we sequentially exclude the 2007–2008 global financial crisis, the 2015 Chinese stock market turbulence, and the post-2020 COVID-19 period. The results are shown in Table 7. The steps are as follows: first, firms registered in municipalities are excluded, with the regression result reported in column (1); second, in addition to municipalities, firms registered in sub-provincial cities are also excluded, yielding the result in column (2); finally, firms in municipalities, sub-provincial cities, and provincial capitals are all excluded, and the result of column (3) is obtained. These results show that, although the statistical significance of the coefficient for fiscal intervention weakens slightly, it remains significantly negative. Excluding high- administrative-level city samples has little impact on the main regression results, confirming that the baseline conclusion holds even within the scope of ordinary prefecture-level cities.

**Table 7:** Robustness check results – exclusion of high-administrative-level cities.

Variable	Explained Variable: *Operating_Assets*		
	Exclude samples of municipalities	Exclude samples of municipalities and sub-provincial cities	Exclude samples of municipalities, sub-provincial cities, and provincial capitals
	(1)	(2)	(3)
*Intervene*	−0.18432***	−0.19816**	−0.20019*
	(−3.22)	(−2.35)	(−2.01)
*Lev*	0.06646***	0.08228***	0.08933***
	(3.99)	(5.07)	(6.72)
*Growth*	0.00123	0.00099	0.00100
	(0.44)	(0.31)	(0.33)
*Size*	−0.00882	−0.01150	−0.01152*
	(−1.14)	(−1.50)	(−1.73)
*ROA*	−0.22198***	−0.21017***	−0.21588***
	(−9.36)	(−5.98)	(−8.09)
*Top1*	0.00021	0.00037	0.00025
	(0.68)	(1.13)	(0.68)
*Msh*	−0.00046***	−0.00044**	−0.00037*
	(−3.18)	(−2.27)	(−1.94)
*Board*	−0.00190	−0.00168	−0.00044
	(−1.34)	(−1.01)	(−0.28)
*Ins*	−0.00030*	−0.00035*	−0.00029*
	(−1.84)	(−2.03)	(−1.79)
*Lnpergdp*	0.01580	0.00733	0.00045
	(1.19)	(0.41)	(0.03)
*Industry*	0.01220	0.02755	0.03934
	(0.29)	(0.53)	(0.83)
*Market*	−0.00446	−0.00480	−0.00608
	(−0.96)	(−0.87)	(−1.17)
*Debt*	0.02534	0.03885*	0.00384
	(1.27)	(1.92)	(0.15)
*Sale*	−0.02609***	−0.02638**	−0.01505
	(−2.73)	(−2.47)	(−1.31)
*Constant*	0.83146***	0.94741***	0.83469***
	(4.85)	(4.75)	(3.81)
Firm/Year-Level Fixed Effects	YES	YES	YES
*N*	19546	13116	10632
*adj. R* ^*2*^	0.743	0.745	0.737

Note: *, **, and *** denote significance at the 10%, 5%, and 1% levels, respectively. The t-statistics in parentheses are based on robust standard errors clustered at the two-digit industry level (industry codes). Control variables are lagged by one year.

## 4. Further analysis

### 4.1 Fiscal intervention and corporate financial assets allocation

Having established that fiscal intervention reduces operating asset allocation, we ask: where do the freed resources go? As the resource allocation decision-maker, a firm’s total internal resources are relatively fixed over a given period. When changes in the external environment reduce its willingness to allocate to a certain type of asset, these resources may be reallocated to other types of assets. Operating asset allocation is typically linked to the development of a firm’s core business and long-term value creation, but they have a long investment return cycle and may not generate sufficient short-term cash flow. In contrast, financial assets generally have higher liquidity and can generate short-term returns in certain market conditions. Therefore, when faced with pressure from prefecture government economic intervention, firms may form a tendency to move more resources from operating assets to financial assets to pursue short-term financial gains and engage in speculative arbitrage. In recent years, the phenomenon of corporate financialization has become increasingly prominent in developing and emerging economies, with non-financial corporations allocating more resources to financial assets and deriving a larger proportion of their profits from financial activities rather than productive operations [49,51].

If prefecture government fiscal intervention, by distorting market mechanisms and increasing policy uncertainty, lowers the expected net returns from investing in tangible assets, does it indirectly motivate firms to allocate more resources to the financial sector? To answer this question, we first define the level of corporate financialization (*Financial_Assets*) as the ratio of the sum of held-to-maturity investments, trading financial assets, derivative financial assets, investment properties, financial assets purchased under resale agreements, available-for-sale financial assets, dividends receivable, interest receivable, other non-current financial assets, monetary funds, and long-term equity investments to total assets. We then use *Financial_Assets* as the dependent variable in the regression, with the results shown in column (1) of Table 8. The coefficient for prefecture government fiscal intervention is positive and significant at the 5% level, indicating that the higher the intensity of regional fiscal intervention, the greater the degree of corporate financialization. These empirical results confirm that prefecture government fiscal intervention not only suppresses corporate investment in tangible assets but also induces firms to reallocate more resources toward financial assets. Faced with a less attractive environment for operating investment, firms may invest in more liquid financial assets or those with potential for high short-term returns to secure immediate gains or as a liquidity reserve to meet future funding needs. However, from the perspective of healthy economic development, if this shift in resource allocation bias becomes widespread, it could signal a weakening of real economic vitality and an increase in financial speculation risks.

**Table 8:** Fiscal intervention and corporate financialization.

Variable	Explained Variable: *Financial_Assets*	
	(1)	(2)
*Intervene*	0.17689**	0.01301
	(2.14)	(0.19)
*Intervene*×*H_Real*		0.17883***
		(3.47)
*H_Real*		−0.06362***
		(−9.12)
*Lev*	−0.29514***	−0.24347***
	(−12.69)	(−10.12)
*Growth*	−0.01019***	−0.00757**
	(−3.61)	(−2.62)
*Size*	−0.00569	−0.00646
	(−0.97)	(−1.13)
*ROA*	0.09368***	0.09338***
	(3.92)	(3.65)
*Top1*	0.00003	−0.00007
	(0.13)	(−0.28)
*Msh*	0.00097***	0.00025
	(5.66)	(1.54)
*Board*	0.00050	0.00030
	(0.35)	(0.20)
*Ins*	0.00052***	0.00034**
	(3.89)	(2.24)
*Lnpergdp*	−0.00709	0.00188
	(−0.42)	(0.10)
*Industry*	−0.02239	−0.04846
	(−0.52)	(−1.27)
*Market*	−0.00382	−0.00315
	(−0.99)	(−0.83)
*Debt*	0.01692	0.01977
	(1.03)	(1.13)
*Sale*	0.02043**	0.01310
	(2.23)	(1.36)
*Constant*	0.17178	0.27340
	(0.88)	(1.44)
Firm/Year-Level Fixed Effects	YES	YES
*N*	23531	20670
*adj. R* ^*2*^	0.611	0.641

Note: *, **, and *** denote significance at the 10%, 5%, and 1% levels, respectively. The t-statistics in parentheses are based on robust standard errors clustered at the two-digit industry level (industry codes). Control variables are lagged by one year.

### 4.2 The substitution effect of operating and financial assets

Furthermore, we explore the substitution effect of fiscal intervention on firms’ holding of operating assets and financial assets. We have already established that prefecture government fiscal intervention reduces corporate operating asset allocation. Does reduced operating asset allocation directly drive corporate financialization? If this mechanism holds, then for firms with initially high operating asset ratios, the positive impact of prefecture government fiscal intervention on their financial asset allocation should be more significant, as they have greater capacity to reallocate resources to the financial sector after reducing their operating asset holdings.

To test this substitution effect, we define a dummy variable indicating high operating asset levels, *H_Real*. If a firm’s operating asset ratio in the previous year (*Real*_*t-1*_) is greater than the median across all firms in the same year, *H_Real* takes a value of 1, indicating that the firm belongs to the high operating asset allocation group; otherwise, it is 0, for the low operating asset allocation group. We then construct an interaction term between *Intervene* and *H_Real* (*Intervene*×*H_Real*) and incorporate it into the regression model with *Financial_Assets* as the dependent variable. The results are shown in column (2) of Table 8. The coefficient of *Intervene*×*H_Real* is 0.17883, significant at the 1% level. This result indicates that, compared to firms with initially low operating asset allocation, prefecture government fiscal intervention exerts a stronger positive effect on firms with initially high operating asset allocation, resulting in a more pronounced increase in their financialization level. These empirical findings strongly support the resource reallocation effect between firms’ holdings of operating and financial assets: fiscal intervention drives a resource shift from the real sector to the financial sector.

## 5. Mechanism analysis

The results so far document a robust negative effect of fiscal intervention on operating asset allocation and a corresponding increase in financialization. We now examine the underlying mechanism of transaction cost, which we argue serves as the channel through which fiscal intervention operates.

Transaction cost theory [41–44] holds that markets are not frictionless. Firms incur costs of information search, negotiation, contracting, monitoring, and enforcement when conducting transactions. When these external transaction costs are high, firms adjust their boundaries, governance structures, and investment decisions to economize on these costs. Prefecture government fiscal intervention can raise firms’ external transaction costs through three channels [59–63]. (1) Policy uncertainty. Opaque or unpredictable fiscal programs make it difficult for firms to forecast future government actions, increasing the risk premium on long-term specific investments. (2) Deterioration of contract enforcement. Excessive intervention can undermine the impartiality of contract enforcement, raising the risk of contractual default. (3) Rent-seeking and corruption risks. Where governments control significant resources, high fiscal intervention may be accompanied by rent-seeking, forcing firms to bear additional costs to secure access to resources.

To empirically test the transaction cost mechanism, we use two indirect approaches. Since transaction costs are difficult to observe directly, we examine whether the effect of fiscal intervention varies systematically with firms’ sensitivity to transaction costs, as predicted by theory. If fiscal intervention operates through raising transaction costs, firms that are more vulnerable to transaction cost increases should respond more strongly.

### 5.1 Market power

Market power reflects a firm’s bargaining position in supply chain transactions. Firms with weaker market power possess less bargaining power when negotiating with suppliers and customers and are more exposed to opportunistic behavior. When fiscal intervention deteriorates the transaction environment, weaker firms bear disproportionately higher transaction costs. We therefore expect the negative effect of fiscal intervention on operating asset allocation to be stronger for firms with lower market power.

To test this mechanism, we use Tobin’s Q to proxy for a firm’s market power. This indicator, which reflects the ratio of a firm’s market value to the replacement cost of its assets, effectively captures a firm’s growth potential, profitability, and market recognition. We define Tobin’s Q as the ratio of market value to total assets. We then create a dummy variable *L_TobinQ*, which equals 1 if a firm’s Tobin’s Q in the previous year is below the sample mean, and 0 otherwise. We include an interaction term (*Intervene*×*L_TobinQ*) in Equation (1). The result is presented in columns (1) of Table 9. The coefficient for *Intervene*×*L_TobinQ* is −0.08684, negative and significant at the 5% level. This indicates that when the degree of prefecture government fiscal intervention increases, firms that already lack bargaining power exhibit a stronger negative response in their willingness to allocate resources to operating assets. Fiscal intervention amplifies the uncertainty and potential “hold-up” risks in transactions, making these firms more inclined to reduce their holdings of operating assets.

**Table 9:** Fiscal intervention and transaction cost.

Variable	Explained Variable: *Operating_Assets*	
	(1)	(2)
*Intervene*×*L_TobinQ*	−0.08684**	
	(−2.39)	
*Intervene×H_Intensive*		−0.19463**
		(−2.29)
*Intervene*	−0.12699*	−0.11011
	(−1.84)	(−1.48)
*Lev*	0.04426**	0.07536***
	(2.48)	(4.36)
*Growth*	−0.00012	0.00154
	(−0.04)	(0.54)
*Size*	−0.01248	−0.01525**
	(−1.61)	(−2.30)
*ROA*	−0.19665***	−0.23374***
	(−10.48)	(−12.18)
*Top1*	0.00024	−0.00005
	(0.98)	(−0.20)
*Msh*	−0.00013	−0.00052***
	(−0.88)	(−3.80)
*Board*	−0.00231	−0.00173
	(−1.42)	(−1.19)
*Ins*	−0.00014	−0.00036**
	(−0.90)	(−2.53)
*Lnpergdp*	0.00790	0.01588
	(0.70)	(1.20)
*Industry*	0.04098	0.04299
	(1.06)	(0.92)
*Market*	−0.00447	−0.00438
	(−1.18)	(−0.89)
*Debt*	0.02650	0.03677**
	(1.66)	(2.51)
*Sale*	−0.01917**	−0.03224***
	(−2.11)	(−3.08)
*Constant*	0.83930***	1.05587***
	(4.61)	(5.79)
Firm/Year-Level Fixed Effects	YES	YES
*N*	20356	19187
*adj. R* ^*2*^	0.771	0.722

Note: *, **, and *** denote significance at the 10%, 5%, and 1% levels, respectively. The t-statistics in parentheses are based on robust standard errors clustered at the two-digit industry level (industry codes). Control variables are lagged by one year.

### 5.2 Contract intensity

Industries differ in their reliance on formal contracts. High-contract-intensity industries involve more frequent, complex transactions that depend on detailed contractual specifications [64]. These industries should be more sensitive to changes in the contract enforcement environment. If fiscal intervention raises transaction costs by weakening contract enforcement, firms in high-contract- intensity industries should exhibit a stronger response.

To test this mechanism, we use the measure of industry contract intensity from Nunn (2007) and define a dummy variable, *H_Intensive*, which takes a value of 1 if the contract intensity of a firm’s industry in the previous year is above the sample mean, and 0 otherwise. We then subsequently include an interaction term (*Intervene*×*H_Intensive*) in Equation (1). The result is shown in column (2) of Table 9. The coefficient for *Intervene*×*H_Intensive* is −0.19463, negative and significant at the 5% level. This result indicates that for firms in high-contract-intensity industries, the negative impact of prefecture government fiscal intervention on corporate operating asset allocation is more pronounced. When fiscal intervention leads to a deterioration of the regional business environment, increased uncertainty in contract enforcement, and a rise in overall transaction cost, firms that are highly dependent on complex contractual arrangements find it more difficult to invest in operating assets and are thus more likely to reduce their holdings.

Together, the market power and contract intensity results provide converging evidence that fiscal intervention affects operating asset allocation by raising transaction costs.

## 6. Heterogeneity analysis

### 6.1 Ownership structure

Extant literature indicates that firms with different ownership structures exhibit significant differences in resource access capability, business objectives, governance mechanisms, and sensitivity to the external market environment [65,66]. Private enterprises typically face stronger market competition, with business decisions that are more profit-driven. Meanwhile, without implicit government guarantee or direct support, private firms are more sensitive to changes in the regional market environment. In contrast, state-owned enterprises (SOEs) in China often assume policy-related objectives, such as stabilizing employment and executing industrial policies. They have greater ease in obtaining resources from prefecture governments and, to some extent, enjoy a degree of protection from them. Therefore, we expect that SOEs and private enterprises will respond differently to fiscal intervention, with the negative impact of prefecture government fiscal intervention on corporate operating asset allocation being more significant for private enterprises.

To test for heterogeneity based on ownership structure, we divide the sample into two groups—private enterprises and SOEs—based on the type of their ultimate ownership and estimate the model separately for each group. The results are shown in columns (1) and (2) of Table 10. For private enterprises, the coefficient for prefecture government fiscal intervention is −0.23243 and statistically significant at the 1% level. However, for the SOE sample, the coefficient lacks statistical significance. This result strongly suggests that the negative impact of prefecture government fiscal intervention on corporate operating asset allocation is predominantly observed among private enterprises, while its effect on SOEs is negligible. This supports the argument that fiscal intervention affects asset allocation by increasing external transaction cost, and private enterprises, which are more reliant on the market environment and lack direct government protection, are more severely affected.

**Table 10:** Heterogeneity analysis.

Variable	Explained Variable: *Operating_Assets*			
	Private Enterprises	State-Owned Enterprises	Enterprises with Low Offsite Investment	Enterprises with High Offsite Investment
	(1)	(2)	(3)	(4)
*Intervene*	−0.23243***	−0.12222	−0.17085**	−0.09502
	(−3.16)	(−1.00)	(−2.15)	(−1.10)
*Lev*	0.09408***	0.04196	0.09001***	0.04096
	(4.97)	(1.52)	(4.45)	(1.45)
*Growth*	−0.00312	0.00388	0.00538	−0.00264
	(−0.90)	(0.83)	(1.17)	(−0.84)
*Size*	−0.01602**	0.00290	−0.00909	−0.00989
	(−2.42)	(0.36)	(−1.23)	(−1.09)
*ROA*	−0.19360***	−0.20796***	−0.22320***	−0.19053***
	(−8.33)	(−3.13)	(−6.45)	(−6.36)
*Top1*	0.00051*	0.00003	−0.00027	0.00043**
	(1.92)	(0.08)	(−0.65)	(2.07)
*Msh*	−0.00043***	−0.00071	−0.00068***	−0.00042*
	(−4.25)	(−0.83)	(−3.45)	(−1.92)
*Board*	−0.00188	−0.00142	0.00057	−0.00342
	(−1.39)	(−0.52)	(0.28)	(−1.58)
*Ins*	−0.00048***	0.00000	−0.00025	−0.00031
	(−3.88)	(0.01)	(−1.55)	(−1.59)
*Lnpergdp*	0.02405	−0.00312	0.02285	−0.00457
	(1.64)	(−0.15)	(1.13)	(−0.37)
*Industry*	0.00493	0.07262	0.00828	0.04718
	(0.09)	(0.87)	(0.13)	(0.68)
*Market*	−0.00576*	−0.00075	0.00144	−0.00886**
	(−1.70)	(−0.14)	(0.25)	(−2.09)
*Debt*	0.00979	0.03683	0.03306	0.00644
	(0.41)	(1.54)	(1.33)	(0.33)
*Sale*	−0.02865**	−0.01434	−0.02746*	−0.00951
	(−2.24)	(−1.12)	(−1.87)	(−0.86)
*Constant*	0.95276***	0.50609**	0.73368***	0.79393***
	(3.89)	(2.16)	(2.96)	(4.01)
Firm/Year-Level Fixed Effects	YES	YES	YES	YES
*N*	15025	8460	10392	12799
*adj. R* ^*2*^	0.696	0.790	0.760	0.784

Note: *, **, and *** denote significance at the 10%, 5%, and 1% levels, respectively. The t-statistics in parentheses are based on robust standard errors clustered at the two-digit industry level (industry codes). Control variables are lagged by one year.

### 6.2 Offsite investment

A firm’s dependence on the economy of its registered location is determined by the geographic distribution of its operations. For firms whose main operations are concentrated in their registered location and have a low level of offsite investment, their business activities and resource acquisition are more closely tied to the prefecture-level economic and institutional environment, rendering them more vulnerable to the fiscal intervention of the prefecture government. In contrast, firms with a large number of offsite subsidiaries and a high level of offsite investment exhibit relatively lower dependence on the government of their registered location. They have greater strategic flexibility and more options for resource allocation, allowing them to diversify risks and optimize resource allocation through cross-regional operations, which can mitigate the negative impact of fiscal intervention by their registered location’s prefecture government. Therefore, we expect that the negative impact of prefecture government fiscal intervention on corporate operating asset allocation will be more pronounced for firms with a low level of offsite investment.

To test for this heterogeneity, we use the number of a firm’s offsite subsidiaries to proxy for its level of offsite investment. We divide the sample into the low offsite investment group and the high offsite investment group, based on whether the number of offsite subsidiaries is below or above the median value of all firms in the same year. We then regress separately for each group, and the results are presented in columns (3) and (4) of Table 10. In the low offsite investment group, the coefficient for prefecture government fiscal intervention is −0.17085 and statistically significant at the 5% level. In the high offsite investment group, however, the coefficient lacks statistical significance. This result demonstrates that prefecture government fiscal intervention exerts a more pronounced negative impact on firms with a lower level of offsite investment. If a firm can diversify risks, access high-quality resources, and reduce its dependence on a single region through cross-regional operations, it possesses greater capacity to “vote with its feet” or make internal adjustments when confronted with prefecture government fiscal intervention. Consequently, its operating asset allocation decisions are less affected by negative shocks, whereas firms rooted in the prefecture economy are more directly exposed to the influence of prefecture government actions.

## 7. Conclusion

This study examines how prefecture government fiscal intervention in China affects corporate operating asset allocation decisions, with a focus on transaction cost mechanisms. Our main finding is that higher fiscal intervention significantly reduces firms’ operating asset holdings. This result is robust to instrumental variable estimation and a battery of sensitivity checks. We further find that the reduction in operating assets is accompanied by a corresponding increase in financial asset allocation, revealing a substitution effect from the real economy toward the financial sector. Our mechanism analysis supports the transaction cost explanation: the negative effect of fiscal intervention is stronger for firms with weaker market power and for firms in industries with higher contract intensity, both characteristics that increase sensitivity to transaction costs. Heterogeneity analysis shows that the effect is concentrated among private enterprises and firms with limited geographic diversification.

These findings carry several policy implications. First, fiscal intervention has not only aggregate effects on investment levels but also structural effects on the composition of corporate assets. Policymakers should be aware that excessive intervention may inadvertently push firms away from productive investment toward financial speculation. Second, the results highlight the importance of institutional quality--specifically, stable policy environments and strong contract enforcement--for encouraging long-term productive investment. Improving the predictability and transparency of fiscal policy may be as important as the specific spending levels. Third, our findings suggest that SOEs’ relative insulation from fiscal intervention, while protecting their investment, also reflects their privileged access to resources rather than superior institutional arrangements.

The findings also offer insights for transition economies beyond China. In economies where subnational governments play an active role in resource allocation, such as India, Vietnam, and many Eastern European countries, similar mechanisms may operate. The external validity of our results likely depends on the degree of subnational fiscal autonomy, the strength of formal contract enforcement institutions, and the extent of government involvement in credit allocation. In contexts where these features resemble China’s institutional landscape, fiscal intervention may similarly shape corporate asset allocation through transaction cost channels. Future research could usefully test these predictions using cross-country data or comparative case studies.

## Supporting information

S1 Table Table A1 Correlation Matrix. (DOCX)
