# Supplementary material for: Prefecture government fiscal intervention and corporate asset allocation: The perspective of transaction cost theory
Source: PLoS One. 2026 Jul 1;21(7):e0345478. doi: 10.1371/journal.pone.0345478 (PMC13322524; doi:10.1371/journal.pone.0345478)
Supplement: S1 Table — (DOCX) [file pone.0345478.s001.docx]

Table A1: Correlation Matrix

| Variabe | *Operating Assets* | *Intervene* | *Lev* | *Growth* | *Size* | *ROA* | *Top1* | *Msh* | *Board* | *Ins* | *Lnpergdp* | *Industry* | *Market* | *Debt* |
| --- | --- | --- | --- | --- | --- | --- | --- | --- | --- | --- | --- | --- | --- | --- |
| *Operating Assets* | 1 |  |  |  |  |  |  |  |  |  |  |  |  |  |
| *Intervene* | -0.0101 | 1 |  |  |  |  |  |  |  |  |  |  |  |  |
| *Lev* | 0.250 | 0.00360 | 1 |  |  |  |  |  |  |  |  |  |  |  |
| *Growth* | -0.0459 | -0.0246 | 0.0273 | 1 |  |  |  |  |  |  |  |  |  |  |
| *Size* | 0.210 | 0.106 | 0.474 | 0.0454 | 1 |  |  |  |  |  |  |  |  |  |
| *ROA* | 0.119 | 0.0113 | 0.0289 | 0.0204 | 0.191 | 1 |  |  |  |  |  |  |  |  |
| *Top1* | -0.240 | -0.0525 | -0.345 | 0.0683 | -0.326 | -0.0748 | 1 |  |  |  |  |  |  |  |
| *Msh* | 0.173 | 0.0101 | 0.196 | -0.00710 | 0.281 | 0.0334 | -0.222 | 1 |  |  |  |  |  |  |
| *Board* | 0.192 | 0.0120 | 0.197 | 0.0421 | 0.412 | 0.485 | -0.656 | 0.249 | 1 |  |  |  |  |  |
| *Ins* | -0.144 | -0.0375 | -0.399 | 0.272 | 0.00170 | 0.140 | 0.180 | 0.00810 | 0.112 | 1 |  |  |  |  |
| *Lnpergdp* | -0.230 | 0.0291 | -0.0857 | 0.000100 | 0.0815 | 0.00860 | 0.159 | -0.107 | -0.0700 | 0.0381 | 1 |  |  |  |
| *Industry* | 0.123 | -0.474 | 0.0163 | 0.00850 | -0.150 | -0.0267 | -0.00620 | 0.0284 | -0.0115 | 0.0248 | -0.547 | 1 |  |  |
| *Market* | -0.214 | -0.0375 | -0.140 | 0.00360 | -0.00870 | -0.0581 | 0.221 | -0.189 | -0.149 | 0.0714 | 0.603 | -0.241 | 1 |  |
| *Debt* | 0.0119 | -0.293 | 0.0240 | -0.0152 | 0.0120 | -0.0370 | 0.00230 | -0.0598 | -0.00540 | -0.0126 | -0.00840 | 0.179 | 0.0255 | 1 |
| *Sale* | -0.228 | 0.0378 | -0.0845 | -0.00260 | 0.0890 | 0.00560 | 0.153 | -0.103 | -0.0700 | 0.0333 | 0.981 | -0.597 | 0.580 | 0.00740 |
